# PReS-FINAL-2322: Outcome of kidney transplantation in paediatric patients with ANCA associated glomerulonephritis: a single-center experience

**DOI:** 10.1186/1546-0096-11-S2-P312

**Published:** 2013-12-05

**Authors:** M Twilt, D Noone, W Hayes, P Thorner, S Benseler, R Parekh, R Laxer, D Hebert

**Affiliations:** 1 Rheumatology, The Hospital for Sick Children, Toronto, Canada; 2 Rheumatology, Birmingham Children's Hospital, Birmingham, UK; 3 Nephrology, The Hospital For Sick Children, Toronto, Canada; 4 Pathology, The Hospital For Sick Children, Toronto, Canada

## Introduction

Kidney transplant outcomes for paediatric patients with end stage kidney disease (ESKD) secondary to ANCA GN, particularly granulomatosis with polyangiitis (GPA) and microscopic polyangiitis (MPA) is limited. Adult data suggests similar allograft survival post transplant to other causes of ESKD.

## Objectives

We aimed to describe our experience of kidney transplantation in paediatric ANCA GN patients.

## Methods

We performed a retrospective review of patients with ANCA GN who developed ESKD and were transplanted at the Hospital for Sick Children (HSC) between 2000 and 2012. All patients were diagnosed at HSC and followed until their transfer to an adult center.

## Results

Since 2000 there have been 6 paediatric patients transplanted with ANCA GN (5 MPA). 5 patients were ANCA positive at diagnosis: 1 c-ANCA, PR3 positive and 4 p-ANCA MPO positive. Age at ANCA GN diagnosis was 10.4 ± 4.3 (Mean ± SD) years (range 4.1 to 15.4). eGFR at diagnosis was 14.1 ± 6.2 ml/min/1.73 m^2^. Renal biopsy category was crescentic in 4 and sclerotic in 2 by the new histopathological classification. Initial treatments included: steroids 6 [100%], cyclophosphamide 4 [66.69%] and PLEX 1 [16.67%]. 2 patients had disease relapse within the first 6 months. 4 patients required dialysis at diagnosis (HD) and remained dialysis dependent. All 6 were dialysis dependent by 6 months post diagnosis. Time from ANCA GN diagnosis to kidney transplant (Mean ± SD) was 31 ± 12 months (range 17 - 48 months). All patients received induction therapy and maintenance immunosuppression with prednisone, mycophenolate mofetil, and tacrolimus. Median duration of follow up post transplantation was 3.5 years (range 1.25 - 6.9). eGFR at last follow up was 71.9 ± 34.7 ml/min/1.73 m^2 ^(range 5.7 - 100.5). 1 patient lost her transplant to biopsy-proven, severe acute cellular rejection due to complete non-adherence to medications after 21 months of stable transplant function. No patient had recurrence of vasculitis.

## Conclusion

Short-term patient and allograft survival in paediatric patients with ESKD secondary to ANCA GN is excellent despite aggressive disease, with no recurrence of vasculitis post transplant.

## Disclosure of interest

M. Twilt: None declared, D. Noone: None declared, W. Hayes: None declared, P. Thorner: None declared, S. Benseler: None declared, R. Parekh: None declared, R. Laxer Grant/Research Support from: novartis, D. Hebert: None declared.

